# Solitary forearm steatocystoma simplex

**DOI:** 10.1093/jscr/rjag511

**Published:** 2026-06-26

**Authors:** Faris Aldaghri

**Affiliations:** Anesthesia and Surgery Department, Imam Mohammed Ibn Saud Islamic University (IMSIU), Riyadh 11432, Saudi Arabia

**Keywords:** steatocystoma simplex, pilosebaceous unit, forearm cyst, plastic surgery, lesion excision

## Abstract

Steatocystoma simplex represents a rare, benign adnexal neoplasm derived from the pilosebaceous unit. Forearm acral involvement is uncommon and can be confused with epidermal cyst. A 40-year-old male presented with a 3 × 2 cm, firm, immobile, and asymptomatic forearm mass. Ultrasonography identified a circumscribed cystic structure containing echogenic debris without hypervascularity. Excisional biopsy established steatocystoma simplex that was lined by thin stratified squamous epithelium with an eosinophilic cuticle and attached sebaceous lobules. Resection of the lesion without injury to the capsule performed and long term follow up revealed no recurrence. The atypical dimensions and anatomical site necessitate histopathological differentiation from more common cystic pathologies. Comprehensive surgical resection remains superior to conservative modalities. This rare lesion requires the histological diagnostic confirmation and complete excision ensures long-term cure. Our case presents need to include steatocystoma in differential diagnosis of forearm cystic lesions.

## Introduction

Steatocystomas are benign pilosebaceous neoplasms histologically defined by sebaceous lobules integrated into the cyst wall. They present predominantly as multiplex variants, whereas the solitary form, steatocystoma simplex, is rare. While definitive epidemiological prevalence remains unestablished, onset typically aligns with adolescent sebaceous gland activation, demonstrating no gender or ethnic predilection [1]. Lesions conventionally localize to the trunk, axillae, and groin; acral involvement remains highly atypical. Lacking malignant potential, clinical management is primarily predicated upon cosmetic amelioration, symptomatic relief, or the necessity for histopathological verification [2].

Pathogenesis frequently implicates mutations in the *KRT17* intermediate filament gene. Familial cohorts exhibit autosomal-dominant inheritance occasionally demonstrating phenotypic overlap with pachyonychia congenita type 2. While mutation-negative sporadic cases predominate, definitive diagnosis necessitates histopathological verification [3]. The pathognomonic microscopic signature comprising a thin stratified squamous epithelial lining with an attenuated granular layer, an undulating eosinophilic cuticle, and intramural sebaceous lobules is a requisite for differentiating steatocystoma from epidermoid, vellus hair, and dermoid cysts [1].

## Presentation

A 40-year-old male with an unremarkable medical, surgical, and familial history presented with a solitary, slow-growing, 3 × 2 cm forearm mass. Physical examination revealed a firm, non-tender, immobile lesion lacking a central punctum or overlying skin discoloration. Although asymptomatic, the patient reported significant psychosocial distress regarding the lesion’s visibility.

**Figure 1:**
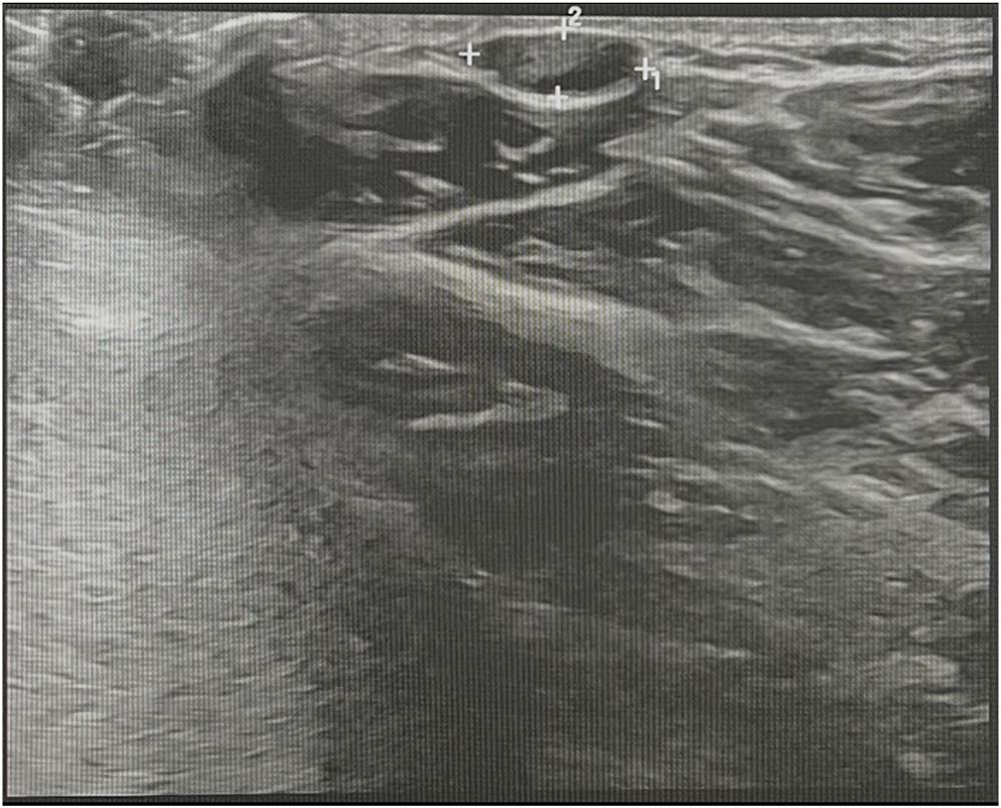
Ultrasound image showing the lesion.

Targeted ultrasonography identified a well-circumscribed, thin-walled cystic structure containing internal echogenic debris. While color Doppler indicated peripheral soft tissue vascularity, there was no evidence of deep fascial penetration or neurovascular compromise. Radiographic findings were consistent with a benign cystic neoplasm (Fig. 1).

### Histopathology

**Figure 2:**
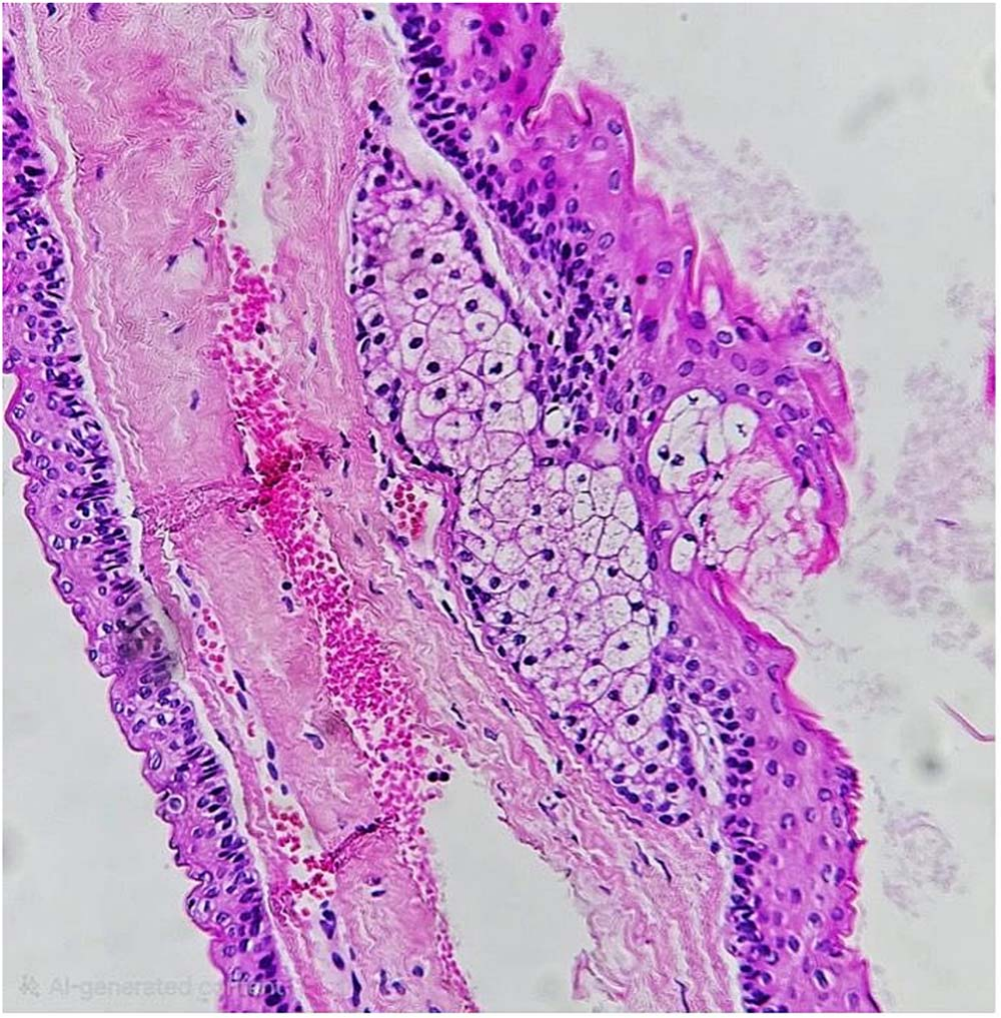
Histopathological section of the lesion.

Core biopsy sample from the lesion revealed a dermal cyst with identical lining. The cyst wall was formed by flattened stratified squamous epithelium. The luminal surface displayed a corrugated, wavy pattern with a prominent eosinophilic cuticle. Focal sebaceous gland lobules were closely opposed to the cyst wall. Luminal material consisted of keratinous debris and sebaceous content. No cellular atypia or mitotic activity was identified on routine hematoxylin and eosin stains. The histological features were characteristic of steatocystoma (Fig. 2).

### Surgical management

Adhering to oncological principles for benign adnexal neoplasms, a 5 mm elliptical incision was demarcated over the ipsilateral forearm lesion. Local anesthesia was achieved via infiltration of 1% lidocaine with epinephrine to facilitate analgesia and regional hemostasis. Emphasis was placed on maintaining capsular integrity to preclude intraoperative rupture. Hemostasis was secured, and the cyst was excised *en-bloc* to eliminate residual epithelial elements. Anatomical layered closure was performed to obliterate dead space and ensure precise tissue approximation.

#### Peri-operative course and follow up

Following a standard postoperative recovery and wound care regimen, the patient underwent an 18-month longitudinal surveillance. Despite the inherent limitations of mid-term follow-up in predicting late recurrence, the clinical assessment at 18 months confirmed optimal cicatrization, preserved functional integrity, and no evidence of local relapse.

## Discussion

This case documents an atypically localized, solitary steatocystoma simplex of the forearm, diverging from the characteristic multifocal truncal distribution. Diagnostic confirmation was achieved via ultrasonographic and histopathological correlation, identified by a stratified squamous lining with integrated sebaceous lobules, thereby excluding epidermal inclusion cysts. Meticulous en-bloc surgical resection remains the definitive therapeutic gold standard; in this instance, preserving capsular integrity ensured total epithelial clearance. Strategic layered closure successfully obliterated dead space, mitigating risks of postoperative seroma or hematoma. The 18-month recurrence-free interval corroborates the efficacy of extracapsular dissection, which historically yields superior outcomes (recurrence rates 3.3%) compared to CO₂ laser ablation (8.3%). Modern minimally invasive excision techniques that prioritize complete capsular removal similarly demonstrate negligible recurrence, reinforcing the necessity of comprehensive cyst wall retrieval for long-term clinical resolution [4].

While minimally invasive modalities, including needle aspiration, cryotherapy, and laser ablation, offer superior cosmetic outcomes, they are historically associated with higher recurrence secondary to retained epithelial remnants. Aspiration and simple expression frequently leave the cyst wall intact, while the efficacy of cryotherapy and incision & drainage techniques remains limited by unpredictable relapse rates. Consequently, for solitary, voluminous, or functionally significant lesions, surgical excision remains the definitive therapeutic gold standard to ensure total capsular clearance [1, 5, 6].

In contrast to historical cohorts (Table 1), this case exhibits distinct clinico-pathological features regarding both scale and anatomical localization. While Brownstein [7] established the histological hallmarks of thirty solitary lesions across the face, trunk, and extremities, longitudinal postoperative data remained sparse. Our 3 by 2 cm forearm cyst significantly exceeds the typical dimensions reported for solitary variants and occupies an atypical distal distribution. Furthermore, while Saravanan and Akthar [8] demonstrated that excision of solitary lesions is both diagnostic and curative, early literature often lacks the long-term surveillance necessary for a robust assessment of recurrence risk.

**Table 1:** Comparative clinical evidence on steatocystoma cases

#	Author	Year	Case type & site	Diagnosis (key features)	Treatment / management	Follow-up / outcome
1	Brownstein MH [7]	1982	Steatocystoma simplex (solitary cyst) – multiple lesions in 30 reported cases (limbs, face, chest)	Solitary dermal cysts in adults without family history; histology showed thin stratified squamous epithelium with eosinophilic cuticle and sebaceous lobules in wall.	Complete surgical excision of each cyst performed	Long-term outcome rarely detailed; recurrence presumed minimal following total excision though systematic follow-up data limited
2	Saravanan & Akthar [8]	2007	Steatocystoma simplex – forehead lesion	Single forehead cyst initially misdiagnosed as dermoid/epidermal; histology confirmed steatocystoma morphology.	Excisional surgery undertaken for diagnostic and curative intent	Short-term postoperative recovery satisfactory; no structured long-term surveillance documented
3	Alqubaisy & Alkhalifah [9]	2016	Steatocystoma multiplex – bilateral forearms in 37-year-old female	Numerous 5–10 mm subcutaneous cysts on both forearms; ultrasonography revealed multiple hypoechoic nodules without familial pattern.	Conservative observational management adopted; dermatologic monitoring only	Lesions remained morphologically stable over five years; no clinical progression or new lesions observed
4	Shin *et al.* [10]	2019	Steatocystoma multiplex – cervical region in 32-year-old male (likely familial)	Multiple bilateral neck nodules; CT/CECT imaging delineated margins; histology typical; dental anomalies indicated ectodermal association.	Surgical excision offered (patient initially declined); focus on radiologic and phenotypic characterization	Follow-up period not explicitly defined; report prioritized diagnostic and imaging features over longitudinal outcome
5	Ha *et al.* [11]	2013	Familial steatocystoma multiplex – trunk/chest of 22-year-old male and father	KRT17 mutation detected; numerous 0.1–0.5 cm cysts distributed across chest; histology consistent with multiplex form.	Selective lesion excision; genetic evaluation prioritized	Pedigree study confirmed hereditary basis; recurrence discussed conceptually though individual lesion outcomes not detailed

Alqubaisy and Alkhalifah [9] characterized bilateral, multifocal forearm cysts that remained stable over a 5-year observational period, suggesting that small, asymptomatic lesions may be managed conservatively. Conversely, the voluminous and symptomatic nature of the current lesion necessitated comprehensive capsular excision. The integration of advanced imaging as advocated by Shin *et al.* [10] for defining margins in complex cervical presentations parallels our use of preoperative ultrasonography to mitigate neurovascular risk. Furthermore, while Ha *et al.* [11] elucidated the molecular pathogenesis of familial variants via *KRT17* mutation identification, our case underscores the clinical management of sporadic, macro-cystic phenotypes.

This case reinforces the consensus that localized, voluminous solitary cysts achieve a durable cure via meticulous, capsule-intact excision. Such an approach not only ensures a minimal recurrence profile but also facilitates the definitive verification of a sporadic, non-syndromic etiology.

## Conclusion

Solitary steatocystoma simplex of the forearm represents an atypical clinical presentation that frequently parallels common benign adnexal neoplasms. Pathognomonic identification hinges upon the histopathological evidence of a thin stratified squamous epithelial lining with contiguous sebaceous lobules, facilitating differentiation from epidermoid inclusion cysts. En-bloc surgical resection remains the therapeutic gold standard, providing superior functional and aesthetic outcomes alongside minimal recurrence risk.

In this report, preoperative sonographic localization and atraumatic capsule-intact dissection ensured total clearance and an unremarkable postoperative course. The 18-month recurrence-free interval validates the curative necessity of meticulous surgical technique. Consequently, a high index of clinical suspicion is warranted for cystic lesions in aberrant sites, where histopathological confirmation is paramount to avert diagnostic errors or suboptimal management.
